# 24-years’ experience of peritoneal dialysis in a university hospital dialysis center: Management and outcome 

**DOI:** 10.5414/CNP104S06

**Published:** 2025-11-28

**Authors:** Robert Ekart, Tina Stropnik Galuf, Benjamin Dvoršak, Tadej Zorman, Maša Knehtl, Eva Jakopin, Nina Vodošek Hojs, Martin Hren, Nejc Piko, Renata Smogavec, Sebastjan Bevc, Radovan Hojs, Matjaž Horvat

**Affiliations:** 1 Department of Dialysis, Clinic for Internal Medicine, University Medical Center Maribor,; 2 Faculty of Medicine, University of Maribor,; 3 Department of Nephrology, Clinic for Internal Medicine, University Medical Center Maribor, Maribor,; 4 OptimaMed Vojnik, Vojnik, and; 5 Department of General and Abdominal Surgery, Clinic for Surgery, University Medical Center Maribor, Maribor, Slovenia

**Keywords:** peritoneal dialysis, discontinuation, peritonitis, outcome

## Abstract

Introduction: The aim of our study was to analyze the results of peritoneal dialysis (PD) treatment in all patients treated with PD in our dialysis center. Materials and methods: This is a retrospective observational study in which we included all PD patients from the start of PD treatment in 2000 until the end of follow-up on 30 September 2024. Results: During an observation period of 24 years, 89 patients started PD treatment. Two patients who started PD treatment due to heart failure were excluded. The mean age of the patients was 47 ± 14 years, 61 (70.1%) were men. During PD treatment, 30 (34.5%) patients developed PD-related peritonitis after a median PD duration of 1,174 ± 936 days. During follow-up, 27 (31%) were converted to hemodialysis, 24 (27.6%) received a kidney transplant, 15 (17.2%) patients died during PD treatment, and the remaining 21 patients were still being treated with PD at the end of follow-up. The median duration of PD for all patients was 1,414 ± 1,253 days. Cardiovascular disease (50%) and infection (50%) were the most common causes of death among patients. The comparison between surviving and deceased PD patients showed that surviving patients had a higher baseline serum albumin level (37.7 vs. 34.5 g/L; p = 0.004). The Kaplan-Meier survival analysis showed a worse outcome for diabetics (log rank (Mantel-Cox) = 5.457; p = 0.019). Conclusion: Cardiovascular disease and infection were common causes of death in PD patients. Peritonitis was the most common cause of PD discontinuation. Diabetics undergoing PD had a poorer survival rate. The average duration of PD treatment was 3.9 years. The average time until the first peritonitis was 3.2 years.

## Introduction 

Peritoneal dialysis (PD) is an effective therapy for end-stage kidney disease (ESKD) that can improve quality of life and save costs compared to hemodialysis (HD) [1]. PD is favored in newly diagnosed ESKD patients due to its ease of technique, home-based therapy, and better preservation of residual renal function [2]. The choice of dialysis modality should be made after a pre-dialysis education process, taking into account patient characteristics, needs, and preferences. In many countries, PD is under-prescribed compared to HD, and only about 10 – 20% of patients with ESKD worldwide undergo this treatment [3, 4]. In several countries such as China, Hong Kong, Thailand, and the USA, a significant increase in PD utilization has been reported in recent years due to adjusted reimbursement and healthcare policies [1]. 

In Slovenia, relatively few patients decided for PD treatment, so our country’s proportion of PD patients in all three ESKD treatment modalities is below 10%. In our dialysis center, we introduced PD in 2000. The aim of this study was to present the results of the treatment outcomes of all patients who started PD in our dialysis center. 

## Materials and methods 

### Study design and patient recruitment 

We conducted a retrospective study at a single tertiary center. Uremic patients who had a PD catheter implanted and started PD therapy between 2000 and 2024 at the Department of Dialysis, Clinic for Internal Medicine, University Medical Centre Maribor, Slovenia, were recruited. All patients were followed until transferring to HD, kidney transplantation, death, or until 30 September 2024. Patients were categorized into 4 groups (≤ 2 years, 2 to ≤ 5 years, 5 to ≤ 10 years, or > 10 years) based on dialysis duration in September 2024. 

### Clinical data 

Baseline demographic and clinical data such as age, sex, primary kidney disease, comorbidities, initial PD modality, serological parameters, etc. were collected. All patients were dialyzed with a glucose-based PD solution with a dextrose/glucose concentration of 1.36, 2.27, or 3.86% (Dianeal or Physioneal (Baxter Healthcare Corporation, Deerfield, IL 60015 USA)) and icodextrin. Dialysis-related parameters were collected during the first peritoneal equilibration test (PET), which was performed 1 – 6 months after PD started. 

PD-related infections included peritonitis as well as exit-site and tunnel infections. The diagnosis of PD-associated peritonitis required two of the following: clinical features such as abdominal pain or cloudy dialysis effluent; white blood cell count > 100/μL in the dialysis effluent (after a dwell time of at least 2 hours), with > 50% neutrophils; and a positive culture of the pathogen in the dialysis effluent [5]. 

Cardiovascular disease during PD treatment was defined as acute coronary syndrome, heart failure, stroke, heart valve replacement, coronary artery bypass grafting, hypertensive encephalopathy, or peripheral vascular disease. 

The study was conducted in accordance with the Declaration of Helsinki. Due to the retrospective and observational nature of the study, informed consent was not obtained for this study. The study was approved by the Committee for Medical Ethics at the University Medical Center Maribor (UKC-MB-KME-18/25). 

### Statistical analysis 

Continuous variables are presented as mean and standard deviation (mean ± SD), categorical data as frequencies and percentages (n, %). Student’s T-test for independent samples was used to compare two groups. The estimated survival probability of patients was analyzed using the log-rank test and presented using the Kaplan-Meier curve. Statistical significance was considered as p < 0.05, and all probability tests reported were two-sided. Statistical analysis was performed using IBM SPSS software, version 29.0.0.0 (IBM, Armonk, NY, USA). 

## Results 

During an observation period of 24 years, 89 patients started PD treatment. In 3 patients the PD catheter was implanted in another medical institution, while in all other patients it was inserted in our medical center. Two patients who started PD treatment due to heart failure were excluded, so we analyzed the treatment of 87 patients. The mean age of the patients was 47 ± 14 years, 61 (70.1%) were men. The most common causes of ESKD were diabetes (25.3%), hypertension (21.8%), IgA glomerulopathy (17.2%), other types of chronic glomerulonephritis (12.6%), and polycystic kidney disease (6.9%). The baseline demographics of the patients at the start of PD treatment are shown in Table 1. 

**Figure 1:**
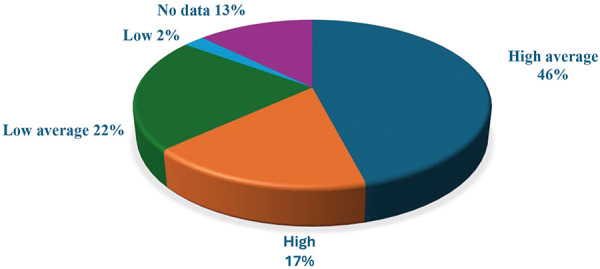
First peritoneal membrane equilibration test – membrane type classification: N = 87.

Before the start of PD treatment, 28 (32.2%) patients were treated with HD. One year after starting PD treatment, 56% of patients were treated with CAPD, 32% with automated PD (APD), and 11% discontinued PD. The results of the first PET test are presented in Figure 1. 

**Figure 2:**
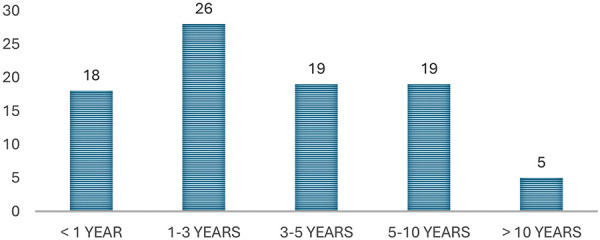
Whole duration of peritoneal dialysis treatment for all patients (N = 87).

26 (29.9%) patients were treated with PD for 1 – 3 years, 19 (21.8%) patients for 3 – 5 years, 19 (21.8%) patients for 5 – 10 years, 18 (20.7%) for less than 1 year, and 5 (5.7%) patients for longer than 10 years (Figure 2). The longest period of PD treatment in 1 patient was 7,329 days (20.1 years). The median duration of PD in all patients was 1,414 ± 1,253 days (3.9 ± 3.4 years; 43 – 7,329 days). 

During follow-up, 27 (31%) patients were converted to HD and 24 (27.6%) received a kidney transplant. The most common causes of conversion from PD to HD were peritonitis in 11 (40.7%) patients, hypervolemia and ultrafiltration failure in 7 (25.9%) patients, and misplacement of the PD catheter in 3 (11.1%) patients. 15 (17.2%) patients died during PD treatment. Cardiovascular disease (50%) and infections (50%) were the most common causes of death among patients. During PD treatment, 30 (34.5%) patients developed PD-related peritonitis (mean peritonitis rate 1 episode/136.7 PD months or mean peritonitis rate of 0.09 per patient-year) and 12 (13.8%) developed exit-site infection. Four (4.6%) patients died during peritonitis episode. The median PD duration until the first PD-related peritonitis was 1,174 ± 937 days (3.2 ± 2.6 years). A statistically significant difference in serum albumin (mean 35.6 vs. 38 g/L, p = 0.038) and C-reactive protein (mean 18.1 mg/L vs. 6.6 g/L, p = 0.005) at the start of PD treatment was found between the group of patients with subsequent peritonitis and the group of patients without peritonitis. Factors associated with discontinuation of PD and transfer to HD included a lower age (p = 0.032), lower systolic blood pressure (p = 0.002) and diastolic blood pressure (p = 0.007), and higher leukocytes at the start of PD (p = 0.023) (Table 2). 

The comparison between surviving and deceased PD patients showed that the surviving patients had a higher baseline serum albumin level (38 vs. 35 g/L; p = 0.03) and leukocytes (7.2 vs 5.7; p = 0.017) (Table 3). 

**Figure 3:**
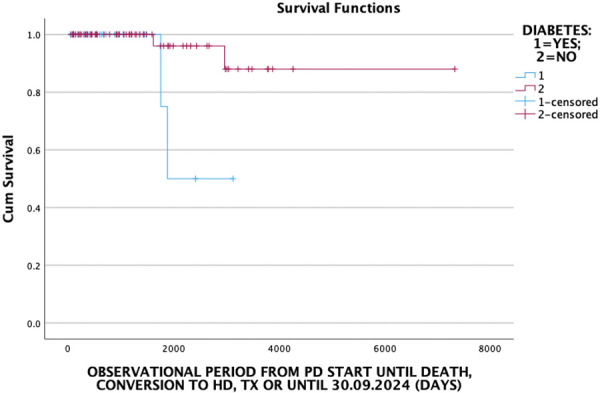
Kaplan-Meier survival curves according to the status of diabetes.

The Kaplan-Meier survival analysis showed that diabetic patients had a poorer survival outcome (log rank (Mantel-Cox) = 5.457; p = 0.019) (Figure 3). 

## Discussion 

Treating patients with PD for as long as possible is undoubtedly a major challenge, especially because of the many factors that can influence treatment outcome and lead to premature PD discontinuation. In our single-center study, we analyzed data from a cohort of 87 PD patients to investigate their outcomes and risk factors for PD discontinuation. We found that the average duration of PD treatment for all patients was 3.9 years, and the average time to first peritonitis was 3.2 years. 

PD patients who were alive at the end of follow-up had a higher serum albumin and leukocytes at the start of treatment. In a study from the Netherlands (NECOSAD database), the risk factors for discontinuation of PD were also those responsible for patient survival: age, cardiovascular disease, diabetes, and residual glomerular filtration rate [6]. In general, young patients had better overall health than older patients, and they stayed on PD for a long time [6]. On the other hand, older patients had a variety of comorbidities and complications that increased the carryover to HD. 

In our study, patients with diabetes as the main cause of ESKD had a worse outcome compared to non-diabetic patients. The mean duration of treatment with PD was 985 ± 788 days in diabetic patients compared to 1,559 ± 1,349 days in non-diabetic patients. The prevalence of diabetes has been reported to be an independent predictor of poor patient and technique prognosis in PD patients, as these patients had notable comorbidities [7, 8]. In a study by Xie et al. [9], in a cohort of 586 PD patients, they found that long-term PD patients were younger, had a lower prevalence of diabetes, better nutritional status, no inflammation, better residual renal function, and more frequent use of renin-angiotensin system inhibitors at baseline. 

In a retrospective study by Isla et al. [10], malnutrition was a marked risk factor associated with both patient death and technical failure in patients undergoing PD. Serum albumin, hemoglobin concentration, and more than 1 episode of peritonitis were the factors that predicted the overall outcome [10]. In our study, we found statistically significantly lower baseline albumin levels in the deceased patients, which confirms some role of malnutrition in the outcome of our patients. 

In a recently published meta-analysis of 72 studies involving more than 630,000 patients, the survival benefit of early nephrologist referral in patients prior to dialysis was demonstrated irrespective of dialysis modalities [11]. Patients who were referred earlier had a shorter initial hospitalization and were better prepared for renal replacement therapy [11]. Unfortunately, we did not analyze the timing of referral to the nephrologist in the patients included in our study, so we cannot comment on the reason for starting PD at very low estimated glomerular filtration rate (eGFR) values. 

One of the most important tasks of nephrological care is patient education. Since 2015, we have been offering individual pre-dialysis education at our dialysis center for all patients with chronic kidney disease who reach an eGFR below 20 mL/min/1.73m^2^ for the first time. This type of education is only provided by nurses with knowledge of PD. In this way, we were able to significantly increase the number of PD patients compared to the group education of patients in previous years, which was conducted by all dialysis nurses and not only by PD nurses. 

The results of our study showed a very low peritonitis rate (the mean peritonitis rate was 0.09 per patient per year) and peritonitis-related mortality (4.6%). This result is due to enhanced care management by nurses, who are an extremely important part of the overall dialysis team. 

There is growing evidence that patients on PD have comparable (or better) clinical outcomes and a better quality of life than patients on HD [12]. In addition, PD is generally less expensive than HD, making this renal replacement therapy a cost-effective option for ESKD patients. 

There were several limitations in the current study that need to be discussed. First, it is a retrospective study performed at a single center, so the results need to be confirmed by prospective studies. Second, we only had the baseline characteristics of the patients studied, so we were not able to examine the effects of longitudinal changes in clinical parameters on PD outcome. Furthermore, all patients were dialyzed with dextrose/glucose PD solutions, new generations of dialysates, including icodextrin-based solutions, as well as APD using cyclers. Such a diverse group of patients with different variables over such a long period of time may complicate the interpretation of the results. 

In conclusion, peritoneal dialysis is a viable option for renal replacement therapy and can lead to good outcomes as shown in our study. 

## Authors’ contributions 

Conceptualization, R.E. and R.H.; methodology, R.E., S.B., and M.H.; acquisition of patient data, R.E., T.S.G., M.H., B.D., E.J., T.Z., N.V.H., M.K., N.P., and R.S.; validation, R.E.; formal analysis, R.E.; writing – original draft preparation, R.E.; writing – review and editing, all authors; visualization, R.E. All authors read and approved the final version of the manuscript. 

## Funding 

The authors did not receive grants or other funding for this work. 

## Conflict of interest 

The authors have no conflict of interest to declare. 

**Table 1.** Baseline characteristics of patients at peritoneal dialysis initiation (N = 87).

Variable	Minimum	Maximum	Mean ± SD
Age (years)	15	83	47.3 ± 14.4
Residual diuresis (mL/24h)	200	3,000	1,272 ± 635
Serum creatinine (μmol/L)	476	1290	795 ± 169
BUN (mmol/L)	5.4	41.9	26.8 ± 7.2
Estimated glomerular filtration rate (mL/min/1.73m^2^)	3	12	6.2 ± 1.7
Hemoglobin (g/L)	73	138	107 ± 13
C-reactive protein (mg/L)	0	135	10.7 ± 18.5
Serum albumin (g/L)	21	47	37 ± 5
Sodium (mmol/L)	118	144	136 ± 4
Serum potassium (mmol/l)	2.9	6.8	4.7 ± 0.7
Serum phosphorous (mmol/L)	0.9	2.8	1.8 ± 0.5
Alkaline phosphatase (μkat/L)	0.6	6.6	1.5 ± 1.04
Cholesterol (mmol/L)	2	7.8	4.1 ± 1.2
Triglycerides (mmol/L)	0.4	4.5	1.6 ± 0.8

BUN = blood urea nitrogen.

**Table 2.** Factors associated with peritoneal dialysis withdrawal and transfer to HD.

Variable at PD initiation	Without transfer to HD (N = 37)	Transfer to HD (N = 27)	p-value
Age (years)	53 ± 14	45 ± 13	0.032
Systolic BP (mmHg)	156 ± 20	141 ± 14	0.002
Diastolic BP (mmHg)	94 ± 12	86 ± 11	0.007
Residual renal function (mL/24h)	1,266 ± 519	1,291 ± 782	0.881
eGFR (mL/min/1.73m^2^)	5.9 ± 1.5	6.4 ± 1.6	0.291
Hemoglobin (g/L)	107 ± 11	104 ± 15	0.332
CRP (mg/L)	12.3 ± 24.2	10.9 ± 14.6	0.802
Leukocytes (10^9^/L)	6.2 ± 1.7	7.4 ± 2.4	0.023
Albumin (g/L)	36.9 ± 4.9	36.5 ± 5.4	0.735
Follow up until the end of study (days)	1,571 ± 1,247	1,317 ± 1,438	0.455

BP = blood pressure; CRP = C-reactive protein; eGFR = estimated glomerular filtration rate; HD = hemodialysis; PD = peritoneal dialysis.

**Table 3.** Comparison of parameters at the start of treatment between surviving and deceased peritoneal dialysis patients (N = 87).

Variable at PD initiation	Dead (N = 15)	Survival (N = 72)	p-value
Age (years)	53 ± 12	46 ± 15	0.106
Systolic BP (mmHg)	154 ± 17	147 ± 21	0.242
Diastolic BP (mmHg)	92 ± 10	90 ± 13	0.524
Residual renal function (mL/24h)	1,313 ± 630	1,263 ± 640	0.783
BUN (mmol/L)	29 ± 8	26 ± 7	0.118
Serum creatinine (μmol/L)	755 ± 142	804 ± 174	0.309
eGFR (mL/min/1.73m^2^)	6.6 ± 1.2	6.1 ± 1.7	0.290
Hemoglobin (g/L)	104 ± 11	108 ± 14	0.306
CRP (mg/L)	12 ± 16	10 ± 19	0.795
Leukocytes (×10^9^/L)	5.7 ± 1.1	7.2 ± 2.4	0.017
Albumin (g/L)	35 ± 6	38 ± 5	0.03
Cholesterol	4.5 ± 1.2	4 ± 1.1	0.147
Triglycerides	1.9 ± 1	1.6 ± 0.8	0.184

BP = blood pressure; BUN = blood urea nitrogen; eGFR = estimated glomerular filtration rate; CRP = C-reactive protein.
